# Head Injury Exposure in Veterans Presenting to Memory Disorders Clinic: An Observational Study of Clinical Characteristics and Relationship of Event-Related Potentials and Imaging Markers

**DOI:** 10.3389/fneur.2021.626767

**Published:** 2021-06-14

**Authors:** Katherine W. Turk, Anna Marin, Kylie A. Schiloski, Ana L. Vives-Rodriguez, Prayerna Uppal, Cheongmin Suh, Brigid Dwyer, Rocco Palumbo, Andrew E. Budson

**Affiliations:** ^1^Center for Translational Cognitive Neuroscience, VA Boston Healthcare System, Boston, MA, United States; ^2^Alzheimer's Disease Research Center, Boston University, Boston, MA, United States; ^3^Department of Neuroscience, Boston University, Boston, MA, United States; ^4^Department of Psychological, Health, and Territorial Sciences, D'Annunzio University of Chieti-Pescara, Chieti, Italy

**Keywords:** traumatic brain injury, repetitive head impacts, event relate potential, chronic traumatic encephalopathy, Alzheimer's disease

## Abstract

**Objective:** Traumatic brain injury (TBI) and repetitive head impacts (RHI) related to blasts or contact sports are commonly reported among military service members. However, the clinical implications of remote TBI and RHI in veterans remains a challenge when evaluating older veterans at risk of neurodegenerative conditions including Alzheimer's disease (AD) and Chronic Traumatic Encephalopathy (CTE). This study aimed to test the hypothesis that veterans in a memory disorders clinic with remote head injury would be more likely to have neurodegenerative clinical diagnoses, increased rates of amyloid PET positivity, higher prevalence of cavum septum pellucidi/vergae, and alterations in event-related potential (ERP) middle latency auditory evoked potentials (MLAEPs) and long latency ERP responses compared to those without head injuries.

**Methods:** Older veterans aged 50–100 were recruited from a memory disorders clinic at VA Boston Healthcare system with a history of head injury (*n* = 72) and without head injury history (*n* = 52). Patients were classified as reporting prior head injury including TBI and/or RHI exposure based on self-report and chart review. Participants underwent MRI to determine presence/absence of cavum and an ERP auditory oddball protocol.

**Results:** The head injury group was equally likely to have a positive amyloid PET compared to the non-head injury group. Additionally, the head injury group were less likely to have a diagnosis of a neurodegenerative condition than those without head injury. P200 target amplitude and MLAEP amplitudes for standard and target tones were decreased in the head injury group compared to the non-head injury group while P3b amplitude did not differ.

**Conclusions:** Veterans with reported remote head injury evaluated in a memory disorders clinic were not more likely to have a neurodegenerative diagnosis or imaging markers of neurodegeneration than those without head injury. Decreased P200 target and MLAEP target and standard tone amplitudes in the head injury group may be relevant as potential diagnostic markers of remote head injury.

## Introduction

The relationship between head injury and subsequent dementia, two highly common and often co-occurring neurologic disorders, has gained increased attention in recent years with mounting pathological and epidemiological evidence indicating that head injury likely plays a causal role in the development of chronic traumatic encephalopathy (CTE) (1–3) and may act as a significant risk factor for Alzheimer's disease (AD) (4–8). Less well-understood, however, is the necessary amount and severity of exposure to head injury which leads to increased dementia risk, ranging widely from sub-concussive or concussive repetitive head impacts (RHI) to mild, moderate and severe traumatic brain injury (TBI). While the strongest links are those between RHI and CTE pathological diagnosis (3), epidemiological studies have also found an association between TBI (including mild TBI, mTBI) and increased risk of dementia among both civilian (9) and veteran populations (10–14).

However, the relationship between mTBI and dementia remains unclear and not without controversy (15–17) as a competing line of evidence supports the theory that mTBI injuries resolve within ~3 months without lasting cognitive sequelae (18). Thus, the translation of pathological and epidemiological evidence into clinically meaningful and impactful diagnostic and prognostic information for older individuals with both cognitive complaints and TBI/RHI exposure is an active area of investigation.

The nature, severity and frequency of head injuries are clinically heterogeneous among veterans who frequently have both contact sport and military exposures to head injury. According to prior literature, most individuals with reported RHI also report TBI exposure (19). Furthermore, in many instances the existing TBI literature has failed to account for RHI exposure, which is common in civilian and military settings and may represent an independent and/or synergistic risk factor for neurodegenerative disease.

Amyloid-ß (Aß) is a well-established AD biomarker as it is thought to play a significant role in pathogenesis (20). Positron emission tomography (PET) tracers targeted to Aß including 18F-AV-45 (Florbetapir) have clinical utility in the evaluation of patients with suspected AD (21). As such we hypothesized that veterans in the memory disorders clinic with head injury would show increased rates of amyloid PET positivity.

The presence of a cavum septum pellucidum and/or cavum vergae has been proposed as a potential biomarker of RHI/TBI (22). During head trauma, fluid waves may produce fenestrations within the septum pellucidum or vergae and allow the entry of fluid between the leaflets, creating the cavum within the structure. We evaluated structural imaging studies for all patients in the current study to determine the relative prevalence of a cavum among older veterans in a memory disorders clinic with and without prior head injury exposure. We hypothesized that veterans with a reported history of TBI and/or RHI would also have a relatively increased prevalence of cavums and that this imaging finding may be predictive of an ultimate clinical diagnosis of neurodegenerative condition of any etiology.

As an additional potential biomarker of prior head injury, the current study explored differences in event-related potentials (ERPs) between veterans with and without a history of head injury with the general hypothesis that specific ERP components may allow separation of the long-term cognitive effects of head trauma from those related to neurodegeneration. An auditory oddball paradigm consisting of an infrequent auditory target stimulus while participants are asked to avoid responding to more frequent distractor and standard stimuli (23) was used to evaluate specific middle and longer latency ERP components. ERPs measure electrical activity of the brain in response to external stimuli with a high degree of temporal resolution, are non-invasive, low-cost, and represent a potential electrophysiologic biomarker for evolving brain injury and recovery.

Sensory ERP measures are partly comprised of the Middle latency auditory evoked potentials (MLAEPs) including the P50 waveform occur 10–60 milliseconds following stimulus onset and reflect auditory cortical functioning with generators including the thalamus, inferior colliculus, and primary and secondary auditory cortices (24). Prior ERP investigations of head injury have theorized that abnormal responses may be related to impaired filtering of irrelevant stimuli (25, 26). Therefore, we hypothesized that the MLAEP response to a gating in mechanism (target tones) would display relatively normal MLAEP amplitude and latency, while the response to a gating out mechanism (standard tones) would display a relatively decreased amplitude and increased latency in the head injury group indicating difficulty in filtering out non-target auditory stimuli.

Longer latency ERP measures P200 and P300 components were also evaluated for their ability to reflect possible long-term cognitive deficits related to prior head injury or neurodegeneration in older veterans. The P3b response to target tones occurs between 300 and 500 ms using an auditory oddball paradigm and has been well-investigated in AD where there is decreased amplitude compared to healthy controls (27). P3b amplitude is thought to be related to attentional resource allocation to a working memory task (28), however this peak does not typically show decreased amplitude following head injury (26). We therefore hypothesized that P3b target amplitude would vary with the prevalence of AD pathology within each group, reflecting underlying neurodegenerative disease burden rather than head injury sequalae. Finally, we also investigated P200 amplitude response to target tones between groups with the hypothesis that the head injury group would display decreased P200 amplitude reflecting decreased ability to use attentional resources in target classification. Given that this component has typically not been shown to be altered in AD, compared to healthy control populations (29, 30), we hypothesized that P200 amplitude would be decreased in the head injury group compared to the non-head injury group independently of the degree of AD pathology present in each group.

In addition to these ERP hypotheses, the present study evaluated differences in clinical diagnosis and imaging findings between a group of veterans in a memory disorders clinic with and without reported head injury with the general hypothesis that clinical diagnoses and imaging markers of neurodegenerative disease may be more prevalent among those with head injury. Specifically, we hypothesized that there may be increased rates of both neurodegenerative clinical diagnoses and amyloid PET positivity as well as increased prevalence of cavums among the group with head injuries. We also hypothesized that patients with history of remote head injury would display alterations in both middle and longer latency ERP responses compared to veterans without a reported history of head injury, indicating potential ongoing cognitive impairment related to remote head injury.

## Methods

### Participants and Initial Screening

One hundred twenty-four veterans aged 50-100 were recruited from a memory disorders clinic at VA Boston healthcare system and were categorized regarding their history of head injury (*n* = 72) or absence of head injury (*n* = 52). Head injury status was determined through medical chart review based on a neurologist's assessment at time of initial evaluation as to whether exposure to TBI(s) and/or RHI had occurred at any point in the patient's lifetime as well as complete chart search for the terms “TBI,” “traumatic brain injury,” “concussion,” “head injury,” “football,” “boxing,” and “contact sports.”

TBI was defined using VA/DOD criteria to be a traumatically induced structural injury and/or physiological disruption of brain function as a result of an external force as indicated by new onset or worsening of at least one of the following clinical signs immediately following the event: period of loss of or a decreased level of consciousness, loss of memory for events immediately before or after the injury (posttraumatic amnesia), alteration in mental state at the time of the injury (e.g., confusion, disorientation, slowed thinking, alteration of consciousness/mental state), neurological deficits that may or may not be transient, or intracranial lesion (31). Using current VA/DOD clinical criteria mTBI was defined as TBI with or without loss of consciousness (LOC) where LOC is <30 min, with an alteration of consciousness/mental state lasting between seconds up to 24 h; and if present, period of post-traumatic amnesia lasting up to 1 day. Due to a low number of participants with moderate and severe head injuries, participants with moderate to severe TBI were grouped together and were defined as head injury with LOC > 30 min and alteration in consciousness/mental state beyond 24 h (31). Patients with unknown TBI severity met DOD/VA diagnostic criteria for an mTBI at a minimum, but, given that in some cases presence/absence of a LOC was not recalled, and in other cases the length of LOC could not be recalled, a finer distinction about whether they met mTBI vs. moderate to severe TBI diagnostic criteria could not be made. In keeping with prior RHI literature patients with any reported exposure to contact sports including American football and boxing at the amateur or collegiate level as well as from fights and military exposure to RHI were included within the head injury group (19). RHI may be considered sub-concussive or concussive and in either case patients were included in the RHI sub-group, thus in a majority of instances the RHI group also included individuals with reported prior TBI.

All participants' clinical charts were reviewed for the presence or absence of the following clinical diagnoses at time of first memory disorders clinic evaluation: depression, anxiety, post-traumatic stress disorder, bipolar, hypertension, hyperlipidemia, diabetes mellitus type 2, coronary artery disease, stroke, atrial fibrillation, and OSA.

Participants underwent a non-contrast clinical magnetic resonance imaging study using a 3.0T Siemen's TimTrio scanner with 12-channel head coil located at VA Boston Healthcare System. All images were evaluated by a trained behavioral neurologist (KWT, AVR) who was blinded as to the presence/absence of reported head injury. Coronal and axial T2 Flair images were evaluated to determine the presence/absence of a cavum septum pellucidum and/or vergae. Axial T2/Flair images were reviewed and graded for the degree of white matter chronic ischemic vascular disease using a scale score where 0 = no vascular disease, 1 = mild focal disease, 2 = moderate confluent disease and 3 = severe disease involving U fibers (32). A subset of participants completed a clinical 18F-AV-45 (Florbetapir) amyloid PET scan and the presence/absence of neuritic amyloid plaques was determined by a neuroradiologist who had undergone the appropriate, tracer-specific training.

Participants also completed a neurocognitive test battery that included the Consortium to Establish a Registry in Alzheimer's disease (CERAD) Word List Memory Test (33), Verbal Fluency Test (Category and Letter Fluency) (34), Boston Naming Test-Short Form (35), Trail Making Tests A and B (36), Mini Mental State Examination (MMSE) (37), Geriatric Depression Scale-Short Form (GDS) (38), Geriatric Anxiety Inventory (GAI) (39), and the Center for Neurologic Study-Lability Scale (CNS-LS) (40).

At the time of recruitment, all participants scored 13 or above on the Mini-Mental State Exam (MMSE) (37). All head injured and non-head injured patients were either self-referred for memory complaints or referred to the clinic by their primary care physicians for memory problems. Participants were not excluded based on comorbid conditions or medications, so as to be a representative sample of patients evaluated in a memory disorders clinic.

Between July 2016 and May 2018, a total of 253 participants were consented as part of a larger study evaluating ERPs for the differential diagnosis of dementia in a memory disorders clinic. Of those, 48 were discontinued or excluded because they (i) did not meet the post-EEG inclusion criteria due to low button press accuracy, (ii) had poor audiometry testing, or (iii) experienced problems comprehending and completing the task due to cognitive impairment.

Any patients lacking conclusive documentation of presence or absence of head injury or with reported head injury within 2 years of evaluation were excluded. Out of the 205 subjects that had usable ERP data, an additional 81 subjects were excluded from the data analysis because no conclusive documentation was found regarding the presence or absence of a history of TBI and/or RHI in their medical chart.

### Ethics Approval

Initial human research study approval was granted by the Department of Veterans Affairs, VA Boston Healthcare System Institutional Review Board.

### EEG Testing Procedure

The testing procedure was split into three parts: an initial audiometry test, then a 40 stimuli auditory-oddball practice test, followed by a four-hundred stimuli auditory-oddball full test which spanned ~20-min. All tones were presented through sound-isolating earbuds. Each subject was administered a standard, pure tone audiometry test using the COGNISION System Software. The results from audiometry testing were automatically used by the COGNISION® software to adjust the tone volume to a comfortable level for each participant, thus correcting for any hearing loss. Auditory tone stimuli were amplified to compensate subjects with <30 dB of hearing loss.

Next, participants underwent the auditory-oddball paradigm practice session. Practice session stimuli consisted of 1,000 Hz standard tones (80% frequency) and 2,000 Hz target tones (20% frequency) in pseudorandom order. Participants were instructed to press a button on the handheld set using their dominant hand each time they heard the higher pitched tone. Participants were corrected during the practice test for errors and were allowed to take the practice up to three times to reach at least 80% success. EEGs from the practice session were not used for analysis. The full task consisted of four-hundred frequent standard, infrequent target, and white noise distractor tones appearing with a frequency of 75, 15, and 10%, respectively, in pseudorandom order. The duration for each tone was 100 ms with rise and fall times of 10 ms. The inter-stimulus interval for all the tones was randomized between 1.5 and 2 s. Participants were instructed to press the button as soon as they heard target tones but not for other tones. If participants incorrectly pressed the button for the distractor tone, they were reminded to press the button only for the same tone they had heard in the practice. No other corrections were given during the full task.

### EEG Testing and Data Acquisition

A seven-active electrode COGNISION® EEG rig (Cognision, Louisville, KY) was used to collect ERP data. EEG activity was recorded from sites Fz, Cz, Pz, F3, P3, F4, and P4 sites of the international 10–20 system (41) with reference electrodes on each mastoid process (M1, M2) and one common electrode on the frontal bone (Fpz). The EEG rig was chosen for its ease of use which would allow results to have potentially increased clinical applicability as the rig can be used by clinicians without prior EEG/ERP expertise. The headset used for data collection has been validated to perform reliable ERP recording when skin contact impedance is <70 k Ohms (30). Other work has provided evidence that higher impendence does not affect statistical power when in a cool and dry environment, such as an open office space (42). Impedance was automatically checked at all electrodes and was kept below this limit throughout testing. Data were collected from −240 to 1,000 ms from stimulus onset, digitized at 125 Hz, and bandpass filtered from 0.3 to 35 Hz except for P3b measurements where the low-pass filter was reduced to 16 Hz. An automatic artifact threshold detection limited of +/– 100 uV was set for the tests. Trial sets of a deviant tone and the immediately preceding standard tones (epoch sets) with artifacts exceeding the threshold were rejected in real time and immediately repeated.

Trial averaging and extraction of ERP measures were automatically performed by the COGNISION® System software (Cognision)^19^. EEG data from each trial were baseline corrected using the pre-stimulus period and averaged according to stimulus. For standard tones, only the trials immediately preceding target and distractor stimuli were averaged. During data preprocessing, recording that exceeded two times the root mean square value (RMS) for the EEG test data or with wrong button presses were rejected and excluded from averaging. ERP waves that averaged <20 trials after preprocessing were eliminated from all analyses (30) in keeping with prior literature that recommends a minimum of 16 artifact free trials (43, 44).

P3b area under the curve (AUC) was measured in response to correct target tone trials between for 300–500 ms. P3b measures were obtained from the Pz electrode. P200 AUC was measured in response to correct target tone trials for 150 to 250 ms at the Cz electrode. Time windows were determined based on prior literature and by inspecting individual averages and group grand averages.

P50 peak amplitude was measured as the difference between the mean pre-stimulus baseline and maximum peak amplitude. Peak latency was defined as the time from stimulus onset to the maximum peak amplitude (30). For practical purposes, the P50 ERP feature was defined as the maximum positivity between 24 and 72 ms post-stimulus. AUC was calculated for both standard and target tone MLAEPs in the 10–60 ms time window averaging all electrodes. Accuracy was calculated as the percent of correct responses to target tones (hits) minus button presses to non-targets (false alarms). We replicated the RT calculation methods used by Cecchi et al. (30) as well as in Golob et al. (45). RT is defined as the time from target stimulus onset to release of button press (46). Median Reaction Time (RT) was calculated to limit the influence of any outlier reaction times. Other ERPs and auditory oddball paradigm studies have also employed median RT to perform data analysis (47–50).

After EEG testing, audiometry and button press accuracy results for each participant were further examined using the COGNISION® software to determine cutoffs for inclusion in data analysis. Audiometry scores were scored using hearing thresholds at 1,000 and 2,000 Hz for each ear. These two frequencies correspond to the stimuli used in the auditory oddball paradigm. Patients whose button press accuracy was below 35% on the 400-tone auditory oddball task were excluded from the analysis to preserve reliability of the averaged ERP signals.

### Statistical Analyses

Independent samples *t*-tests were run for comparisons of group means, or Chi-square tests for categorical variables. Binary logistic regressions were conducted to study the relationship between imaging, ERP and clinical variables of interest. Multiple linear regressions were performed to evaluate whether mood and psychiatric disorders as well as vascular risk factors were significant predictors of ERP outcomes of interest. Multiple imputation analyses were performed to account for missing data where appropriate when data was missing at random. Statistical analyses were performed using IBM SPSS version 26.

## Results

As shown in Table 1, there were no significant differences in age or education between the groups (*ps* > *0.0*5). A self-report checklist of depression (GDS) and mood lability (CNS-LS) showed higher levels of depression and mood lability symptoms, respectively, for the head injury compared to non-head injury group (*p* < 0.05). Comparison of neuropsychological testing data between groups demonstrated lower scores for non-head injury patients compared to head injury patients on several neurocognitive tests, including the MoCA and MMSE (*ps*<*0.0*5) as well as on CERAD word list memory encoding and corrected recognition measures where the head injury group was less impaired (*p* < 0.015 where cut-off was corrected for multiple comparisons of CERAD measures).

**Table 1:** Demographic and neuropsychological data for non-head injury and head injury groups.

				**Non-head injured**		**Head injured**	
	***t***	***p***	***Cohens-D***	***N***	**Mean (SD)**	***N***	**Mean (SD)**
Age	−1.72	0.088	0.31	52	73.8 (8.8)	72	71.2 (8.0)
Education	−0.78	0.438	0.15	52	14.2 (2.6)	72	13.8 (2.7)
MoCA^*^	2.35	0.020	1.10	51	19.4 (5.0)	70	24.4 (4.4)
MMSE^*^	2.20	0.030	0.40	52	24.3 (4.0)	72	25.7 (3.0)
CERAD encoding sum^**^	2.99	0.003	0.54	52	12.9 (4.7)	72	15.3 (4.2)
CERAD delayed recall	2.07	0.040	0.36	52	3.0 (2.2)	72	3.8 (2.2)
CERAD recognition	1.72	0.088	0.28	52	8.2 (1.7)	71	8.7 (1.8)
CERAD false positives (FPs)	−2.17	0.032	0.35	52	0.9 (1.8)	72	0.4 (0.9)
CERAD recognition-FPs^**^	2.52	0.013	0.42	52	7.3 (2.6)	71	8.3 (2.1)
GDS^*^	2.73	0.008	0.63	31	3.5 (2.9)	45	5.5 (3.4)
GAI	1.89	0.062	0.44	32	4.8 (5.9)	43	7.4 (5.8)
CNS^*^	3.81	<0.001	1.55	20	9.2 (2.8)	31	13.6 (4.6)
Trails A (time)	−0.95	0.346	0.17	50	62.1 (38.3)	71	55.9 (33.4)
Trails A (errors)	−0.76	0.448	0.18	52	0.2 (0.6)	71	0.1 (0.5)
Trails B (time)	−2.29	0.024	0.42	52	211.8 (91.1)	72	172.5 (96.9)
Trails B (errors)	−1.53	0.131	0.32	33	1.3 (1.3)	54	0.9 (1.2)
FAS (total)	0.33	0.742	0.06	52	31.7 (12.6)	72	32.4 (9.8)
Categories (total)^*^	2.22	0.028	0.40	52	27.7 (12.0)	72	32.1 (10.2)
BNT^*^	2.23	0.027	0.13	52	12.1 (3.0)	71	13.1 (2.1)

*MoCA, Montreal cognitive assessment; MMSE, mini-mental state examination; CERAD, consortium to establish a registry for Alzheimer's disease; GDS, geriatric depression scale; GAI, geriatric anxiety inventory; CNS-LS, center for neurologic study-lability scale for pseudobulbar affect; FAS, phonemic fluency test; BNT, Boston naming test. When participants could not complete Trails B (*n* = 24 for non-head injury group and *n* = 22 for head injury group), score was calculated as equal to 300 seconds*.

*Significant between-group differences:*

* *p < 0.05 and*

** *p < 0.015 CERAD P-values shown are adjusted using the Bonferroni correction for multiple comparisons*.

As shown in Table 2, there was an equal prevalence of cavums in the head injury group compared to the non-head injury group. There was an equal prevalence of cavums in the head injury group compared to the non-head injury group (χ^2^ = 2.32, *p* = 0.13). There was no significant difference in the proportion of positive amyloid PET studies in the non-head injured group compared to the head injured group (χ^2^ = 2.23, *p*= 0.09). There was an increase in the rate of neurodegenerative disease diagnoses in the non-head injured group (73%, 38/52 patients) compared to the head injured group (51%, 37/72 patients) (χ^2^ = 6.05, *p* < 0.02).

**Table 2:** Imaging and clinical findings.

			**Non head-injured**	**Head injured**			
		**Total**			**χ^**2**^**	***df***	***p***
***Cavum***		***N =*** **106**	***N =*** **42**	***N =*** **64**			
	Present	45 (42%)	14 (33%)	31 (48%)	2.32	1	0.13
***Amyloid PET***		***N*** **= 46**	***N*** **= 16**	***N*** **= 30**			
	Positive	19 (41%)	9 (56%)	10 (30%)	2.47	1	0.09
***Neurodegenerative***		***N*** **= 124**	***N =*** **52**	***N =*** **72**	6.05	1	0.014*
***Diagnosis***	Present	75 (60%)	38 (73%)	37 (51%)			

*PET, positron emission tomography*.

Table 3 shows clinical and imaging characteristics of head injury sub-groups. A subgroup analysis comparing the proportion of neurodegenerative diagnoses for mTBI patients to the RHI sub-group revealed that although the RHI group had a numerically greater proportion of neurodegenerative clinical diagnoses (58%, 15/26 patients) compared to the mTBI group (39%, 9/23 patients) this difference was not found to be significant, possibly due to low power (*p* = 0.19). A similar sub-group analysis did not find a greater proportion of positive amyloid PETs in the RHI group (36%, 5/14 patients) compared to the mTBI group (14%, 1/7 patients) (*p* = 0.31).

**Table 3:** Head injury clinical characteristics subgroups.

		**Mild**	**Severe**	**RHI**	**Unknown**
	**Total**				
**Head injury severity**	***N =*** **72**	***N =*** **23**	***N =*** **6**	***N =*** **26**	***N =*** **17**
**# of Head injuries**	***N =*** **72**	***N =*** **23**	***N =*** **6**	***N =*** **26**	***N =*** **17**
1	21 (29%)	10 (43.5%)	5 (83%)	–	6 (35%)
2	4 (6%)	4 (17.4%)	–	–	–
Multiple	38 (53%)	6 (26.1%)	1 (17%)	22 (85%)	9 (53%)
Unknown	9 (12%)	3 (13%)	–	4 (15%)	2 (12%)
**Injury type**	***N =*** **72**	***N =*** **23**	***N =*** **6**	***N =*** **26**	***N =*** **17**
Blunt	30 (42%)	15 (65.2%)	2 (33%)	1 (4%)	12 (70%)
Blast	1 (1.4%)	–	–	–	1 (6%)
Sports	6 (8%)	–	–	6 (23%)	–
Multiple	19 (26.4%)	1 (4.4%)	–	17 (65%)	1 (6%)
Unknown	16 (22.2%)	7 (30.4%)	4 (67%)	2 (8%)	3 (18%)
**TBI vs. RHI**	***N =*** **72**	***N =*** **23**	***N =*** **6**	***N =*** **26**	***N =*** **17**
TBI	46 (64%)	23 (100%)	6 (100%)	–	17 (100%)
RHI	4 (6%)	–	–	4 (15%)	–
Both	22 (30%)	–	–	22 (85%)	–
**Neurodegenerative diagnosis**	***N =*** **72**	***N =*** **23**	***N =*** **6**	***N =*** **26**	***N =*** **17**
No	35 (49%)	14 (61%)	3 (50%)	11 (42%)	7 (41%)
Yes	37 (51%)	9 (39%)	3 (50%)	15 (58%)	10 (59%)
**Amyloid PET status**	***N =*** **30**	***N =*** **7**	***N =*** **2**	***N =*** **14**	***N =*** **7**
Negative	20 (67%)	3 (43%)	2 (100%)	9 (64%)	2 (33%)
Positive	10 (33%)	1 (14%)	–	5 (36%)	4 (57%)

*RHI, repetitive head impacts; PET, positron emission tomography*.

Table 4 details the specific neurodegenerative conditions diagnosed in the head injury and non-head injury groups.

**Table 4:** Neurodegenerative clinical diagnoses.

	**Non-head** **injury**	**Head** **injury**
	***N* = 52**	***N =* 72**
**Mild Cognitive Impairment (MCI)**		
MCI due to AD	7	4
MCI due to DLB	0	2
MCI due to CTE	0	1
MCI due to AD+DLB	1	0
Alzheimer's disease (AD)	23	17
Dementia with Lewy Bodies (DLB)	1	2
Frontotemporal Dementia (FTD)	1	0
Primary age-related tauopathy (PART)	3	3
Dementia due to possible Parkinson's plus	0	1
Possible chronic traumatic encephalopathy	0	2
Dementia possible Parkinson's plus	0	1
Corticobasal degeneration	1	0
Mixed dementia				
* AD+DLB*	1	3
* AD+CTE*	0	1
	38	37

Table 5 details the specific non-neurodegenerative conditions diagnosed in the head injury and non-head injury groups.

**Table 5:** Non-neurodegenerative clinical diagnoses.

	**Non-head** **injury**	**Head** **injury**
	***N =* 52**	***N =* 72**
**Mild cognitive impairment**		
MCI due to mood	5	8
MCI due to chemotherapy	1	2
MCI due to vascular disease	–	3
MCI due to mood and etohism	1	1
MCI due to unclear etiology	2	2
MCI due to mood and substances	1	1
MCI due to untreated OSA and substance abuse	–	1
Vascular dementia	1	1
Subjective cognitive decline	3	11
Normal Pressure Hydrocephalus (NPH)	–	1
Obstructive sleep apnea	–	1
Dementia due to alcoholism or other substances	–	2
Temporal lobe epilepsy related cognitive impairment	–	1
	14	35

### ERP Results

#### Middle Latency ERP Responses

**Figure 1:**
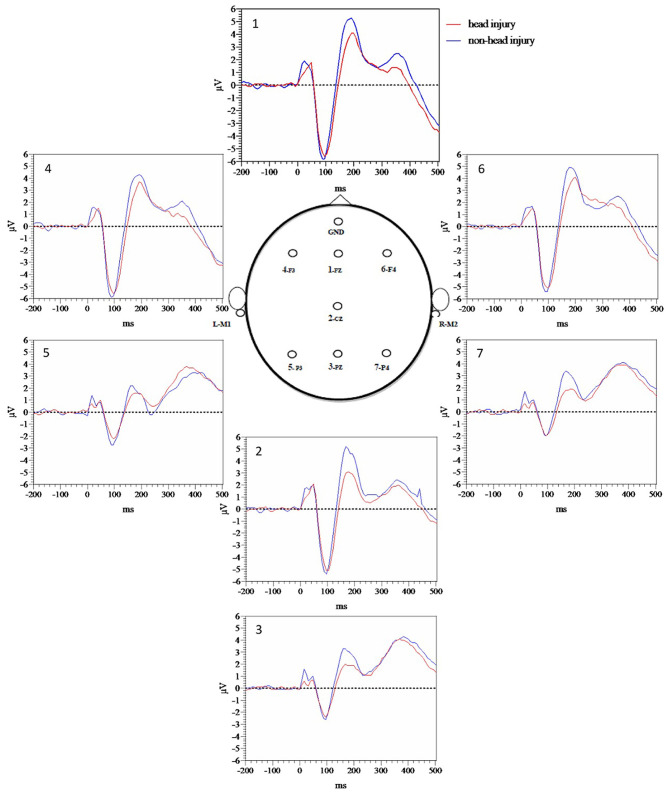
Target tone grand average waveforms for each electrode.

**Figure 2:**
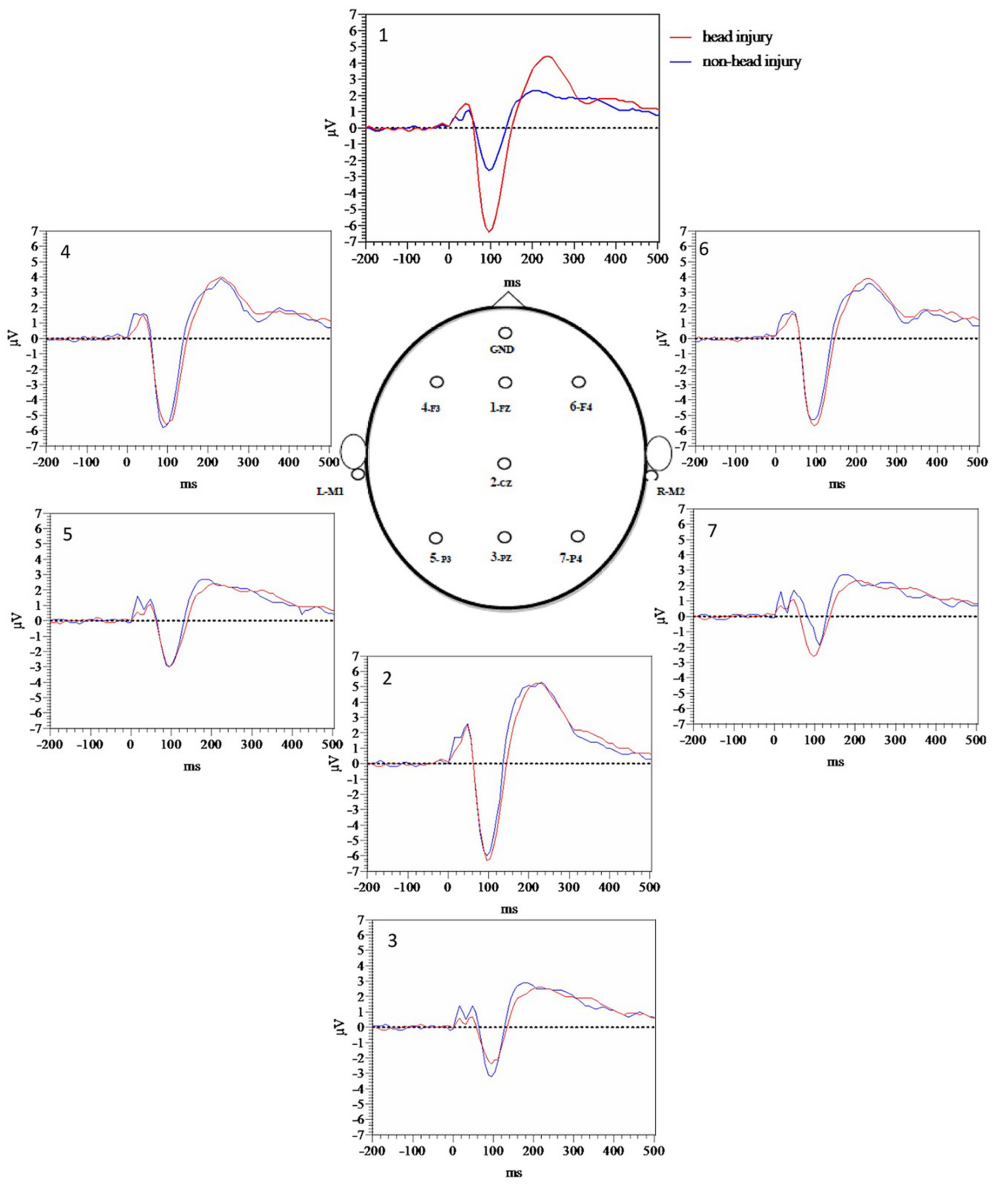
Standard tone grand average waveforms for each electrode.

Figure 1 demonstrates grand average waveforms for each electrode for target tones while Figure 2 demonstrates grand averages for standard tones. Quantitatively, there was a trend toward increased P50 target latency in the head injured group (M = 43.59, SE = 1.79) compared to the non-head injured group (M = 39.0, SE = 1.75), *t*_(122)_ = −1.77, *p* = 0.080). In a subgroup analysis P50 target latency differences between mild and moderate to severe TBI groups revealed that there were no significant differences between the mild (M = 43.9, SE = 2.76) and moderate to severe (M = 41.3, SE = 2.32) TBI groups for P50 target latency [*t*_(36)_ = 0.55, *p* =0.59] with a small number of participants with moderate to severe TBI.

P50 standard latency group differences between all participants with head injury compared to those without head injury demonstrated that while the P50 standard latency for the head injury group (M = 45.2, SE = 1.51) was numerically increased compared to those without head injury (M = 42.6, SE = 1.80), the difference was not significant [*t*_(122)_ = −1.11 *p* = 0.27].

In order to more fully evaluate group differences prior to P50 that appeared visually different based on Figure 1 grand average waveforms for target tones, and Figure 2 grand average waveforms for standard tones we measured area under the curve (AUC) for the 10–60 ms time window with Prism version 9. Averaged target tone AUC was significantly decreased in the head injury group (M = 5.4, SE = 0.71) compared to the non-head injury group (M = 7.57, SE = 0.66, *p* < 0.05). Standard tone AUC was also significantly decreased in the head injury group (M = 5.71, SE = 0.74) compared to the non-head injury group (M = 8.71, SE = 0.71, *p* < 0.05).

#### Long Latency ERP Responses

P3b AUC comparisons between all participants with head injury compared to those without head injury were not significantly different at the Pz electrode (*p* = 0.62). P200 target AUC comparisons between all participants with head injury compared to those without head injury were significantly different at the Pz electrode with decreased P200 target AUC in the head injury group (*p* = 0.02).

#### Binary Logistic Regressions of Amyloid PET Status

In order to determine if ERP responses were predictive of amyloid PET status we completed a series of binary logistic regressions along with age (OR 1.02, 95% CI 0.93–1.12) and education (OR 1.3, 95% CI 0.97–1.69) and found that neither MLAEP AUC for target tones (OR 1.00, 95% CI 0.99–1.01) nor MLAEP AUC for standard tones was a significant predictor (OR 1.00, 95% CI 0.98–1.03). The same was true for P200 target AUC (OR 1.00, 95% CI 0.99–1.002) and P3b target AUC (OR 0.99, 95% CI 0.99–1.001). We also completed a binary logistic regression using age (OR 1.03, 95% CI 0.94–1.13), education (OR 1.27, 95% CI 0.96–1.68) and P50 target latency (OR 0.97, 95% CI 0.93–1.02), none of which were significant predictors of amyloid PET status. Finally we completed a separate logistic regression to determine whether head injury status itself was predictive of amyloid PET results and found that it was not (OR 0.39, 95% CI 0.11–1.35).

#### Binary Logistic Regressions of Neurodegenerative Clinical Diagnosis

We completed a series of binary logistic regressions in order to determine ERP responses were predictive of neurodegenerative clinical diagnosis and found that when run separately in models containing age (OR 1.07, 95% CI 1.03–1.13) and education (OR 1.01, 95% CI 0.87–1.17), neither MLAEP AUC to target (OR 1.00, 95% CI 0.99–1.01) nor standard tones (OR 0.99, 95% CI 0.98–1.01) were significant predictors. Similarly, neither P200 target AUC (OR 1.001, 95% CI 1.00–1.002) nor P3b target AUC (OR 1.00, 95% CI 0.99–1.001) were predictive of neurodegenerative clinical diagnosis. We also completed a binary logistic regression along with age (OR 1.07, 95% CI 1.03–1.13) and education (OR 1.01, 95% CI 0.87-1.17) and found that P50 latency was not a significant predictor of neurodegenerative clinical diagnosis (OR 1.01, 95% CI 0.98–1.03).

#### Binary Logistic Regressions of Head Injury Status

Finally, we completed a separate logistic regression to determine whether head injury status itself was predictive of neurodegenerative clinical diagnosis and found that head injury predicted the absence of a neurodegenerative clinical diagnosis (OR 0.45, 95% CI 0.21–0.944), and age (OR 1.07, 95% CI 1.02–1.13) predicted the presence of a neurodegenerative diagnosis, while education was not a significant predictor (OR 0.99, 95% CI 0.86–1.16). In a separate model presence of a cavum was not a significant predictor of neurodegenerative diagnosis (OR 1.33, 95% CI 0.57–3.1).

#### Multiple Linear Regression Examining Vascular Risk Factor Effects on ERP Measures

Vascular risk factors either individually or in combination including the presence/absence of hypertension, hyperlipidemia, diabetes mellitus type 2, coronary artery disease, obstructive sleep apnea, and stroke were not found to be predictive of any of the ERP responses (Supplementary Tables 1, 2).

Additional linear regression analyses examined the effects of white matter ischemic disease visual ratings as a predictor of ERP outcomes, and found that white matter ischemic disease burden was not a significant predictor of any of the ERP response (Supplementary Table 3).

#### Multiple Linear Regression Examining Mood and Psychiatric Disorders Effect on ERP Measures

The presence of mood disorders either alone or in combination, including anxiety, depression, PTSD and bipolar disorder were not found to be significant predictors of any of the ERP responses (Supplementary Tables 4, 5).

#### Multiple Imputation Analysis

For all missing data, an analysis of missing data and subsequent multiple imputation analysis was completed. A total of 30 variables (61.22% of all variables) were found to contain missing values at a rate of 0.01% or above 0.98 (79.03%) of the cases were missing at least one value and overall there were 281 missing values in the entire data set (4.62%). Among the planned analysis variables cavum and white matter lesions were each missing in 18 cases at a rate of 14.5%. Amyloid PET data missing values showed monotonicity, likely related to the fact that in general participants with amyloid PET ordered clinically were younger. Furthermore, given that PET data was missing at a rate > 50%, sub-group analysis among cases with PET data was performed rather than imputation analysis as supported by prior literature (51).

Although mood, cognitive, and related measures were not part of our final analyses and were included in the demographics section only, scores were missing in 73 cases for CNS-LS, 49 cases for GAI, and 48 for GDS. These measures, which were related to the addition of these questionnaires to the clinical neuropsychological assessment battery used during the study period, and therefore were found to be missing at random. CNS-LS, GAI, and GDS were not part of the main analyses and therefore were not imputed but were used as predictor variables when imputing cavum and white matter lesion variables that were ultimately included in analyses.

Missing data for outcome variables of interest that were missing at random were imputed using SPSS with Markov-Chain Monte Carlo method using all variables and 10 iterations generated five complete data sets that were analyzed individually, the results of which were combined to produce valid estimates. Results did not differ from those produced from data sets without imputed data. More specifically, presence of cavum was not a significant predictor of head injury status or of neurodegenerative diagnosis. White matter lesion burden was not a predictor of MLAEP target or standard tone AUCs, or of P50 target latency. See Supplementary Table 6 for details.

## Discussion

The current study tested the hypothesis that veterans with a history of reported remote head injury in either the form of TBI or RHI would have increased rates of both neurodegenerative clinical diagnoses and amyloid PET positivity compared to those without head injury, and that imaging markers of prior head injury including presence of a cavum would be elevated within the head injured group compared to the non-head injured group. Finally, we hypothesized that P50 target latency, a middle latency auditory evoked measure would be increased in the head injured group as compared to the non-head injured group and may act as a marker of prior head injury.

We found that the prevalence of neurodegenerative clinical diagnoses was actually decreased in the head injured group overall (51%) relative to the non-head injured group (73%) (Tables 2, 4). Although there was a slight trend toward increased P50 target latency among the head injury group compared to the non-head injury group, the differences did not reach significance. However, MLAEP AUC differences revealed that amplitude was decreased in the head injured group compared to the non-head injured group (Figures 1, 2), suggesting that decreased MLAEP amplitude may be a neurophysiological marker of remote prior head injury.

Despite the prevalence of head injury among veterans and its potential impact on life-long cognition, there is a dearth of objective clinical methods available to assess brain function following head injury (52). We had hypothesized that MLAEP amplitude to standard tones would be decreased with increased latency, reflecting a possible inability to effectively filter out irrelevant auditory stimuli. However, we found decreased MLAEP amplitude in the head injury group for both the target and standard MLAEP auditory responses, reflecting a possible decreased ability to detect target stimuli appropriately (impaired gating in), as well as decreased ability to filter out non-target, standard stimuli (impaired gating out mechanism). Furthermore, decreased P200 target AUC was found in the head injury group compared to the non-head injury group as hypothesized potentially reflecting impaired attention during stimulus classification. Finally, P3b amplitude did not differ between head injury and non-head injury groups, which was expected given the finding that there was not an increased rate of neurodegenerative disease in the head injury group and P3b amplitude decreases have been reported in AD populations. The combination of decreases in both MLAEP and P200 amplitude represent a potential advance for the diagnosis of remote head injury among veterans and may allow separation of the long-term cognitive effects of head injury from those of neurodegeneration.

These results are generally in keeping with prior literature regarding ERP findings following head trauma. MLAEP responses are thought to be primarily generated by subcortical and cortical structures throughout the auditory system including the superior olive, inferior colliculus, thalamus, primary auditory cortex, thalamocortical tracts, and lateral supratemporal gyrus and may be modulated by the frontal cortex (24). MLAEPs have been found to be decreased in amplitude in patients with temporal lesions due to strokes when measured 2–4 weeks after injury (53). Prior work has found decreased amplitude in the subacute phase of head injury (54). Others have found increased latency among patients with post-concussive symptoms 48 h after injury, with alterations in MLAEPs remaining at 3 months after injury, but starting to normalize (55). The authors theorized that this trend toward re-normalization after 3 months is unlikely to reflect full return of injured structures to normal function but may be instead due to activation of compensatory tracts for the transmission of the evoked potentials. MLAEP increases in latency at 48 h post-injury were also found to correlate well to degree of subjective neuropsychological impairment 3 months post-injury, suggesting that MLAEPs may offer a physiological measure of diencephalic-paraventricular damage underlying post-concussive symptoms following diffuse axonal injury associated with head injury (55).

The P200 ERP component is primarily generated by the auditory cortex and frontocentral in scalp distribution (56). It is thought to reflect attentional allocation to stimuli inhibitory neurotransmission, thereby affecting temporal processing of stimuli (57). Though prior findings are mixed, a small study in TBI patients found decreased P200 amplitude compared to non-head injured controls in seven patients with moderate to severe TBI chronically after injury (58). The current findings add strength to the hypothesis that P200 amplitude may be decreased long-term following head injury and may reflect ongoing cognitive deficits related to head injury.

It is always possible that ERP outcomes could be attributable to a latent variable associated with head injury that was not measured, however analyses were performed in an attempt to consider potential confounders including vascular disease and psychiatric disease. Middle latency and longer latency ERP responses following remote head injury in an older veteran population from a memory disorders clinic have not been studied previously which underlines the novel nature of the current findings.

No prior studies have investigated MLAEPs following remote head injury among older patients who are presenting with cognitive complaints and at risk for neurodegenerative disease. Results from Soustiel et al. indicate that MLAEPs may begin to normalize within 3 months of injury however were still not at baseline. We observed decreased amplitudes in MLAEPs among older veterans with cognitive complaints and prior head injury. The current findings lead to the possibility that MLAEP abnormalities present earlier on post-head injury could re-emerge in the chronic head injury period with concomitant aging and degeneration of compensatory networks that may otherwise have helped revive MLAEP signals in the subacute injury period.

In order to further evaluate the possible contributions of vascular disease to ERP findings including MLAEP and P200 AUC decreases we performed regressions including the presence of vascular risk factor diagnoses as potential predictors of ERP responses and none of the vascular risk factors examined were found to be significant predictors of the ERP findings. Furthermore, blinded visual assessments of white matter ischemic disease load did not predict ERP results either. Future studies may benefit from diffuse tensor imaging techniques to investigate axonal injury that may underlie MLAEP changes in the head injured group but may not be directly visible using clinical MRI sequences.

It is worth noting that while decreases in amplitude and AUC for several of the ERP components investigated including MLAEP and P200 are typically linked to deficits in cognitive function, the head injury group in the current study showed decreased P200 and MLAEP AUC without corresponding deficits in cognitive testing. While it may be that the current neuropsychological battery is not fully sensitive to the cognitive deficits sustained by head injury patients and their deficits this seems less likely as the battery used well-established measures of domains thought to be affected by TBI including robust measures of executive function. Similarly to our current findings, other groups have have reported that middle latency ERP responses did not correlate well with neuropsychological measures and they hypothesized that changes in electrophysiology may not be fully represented by neuropsychological instruments which may be less fine-tuned to changed in brain structure than EEG methods (59). The discordance between ERP and neuropsychological measures is potentially of great clinical interest if ERPs are able to provide an alternative methodology for following cognition many years after head injury.

While prior findings in the AD population have reported decreased P300 amplitude (30) and increased P50 standard latency compared to controls (60) the current study did not find significant differences between groups. That may be due to the different population in the current study which is relatively unique and includes memory disorders patients with and without head injury.

When comparing the head injured group's neuropsychological results and clinical diagnosis results to those of the non-head injured group, the head injured group was less impaired overall on cognitive neuropsychological measures, consistent with their being less likely to be diagnosed with a neurodegenerative disease. While these findings were not expected given our hypotheses based on epidemiological evidence indicating that even mTBI can impact future risk of dementia (13), there could be a number of explanations.

Prior epidemiological studies that found increased rates of dementia among veterans with head injury compared veterans with TBI to the general population (13). Our findings likely diverge from these prior results given the differences between the memory disorders clinic and the general population—namely that patients in the memory disorders clinic already display greatly increased rates of neurodegenerative disease simply by being referred to the clinic. Patients in our clinic population may have cognitive deficits that fit into one of three possible categories of interest in this study: (1) deficits due to head injury alone in the absence of neurodegeneration, (2) neurodegenerative disease alone in the absence of head injury exposure, and (3) head injury in conjunction with neurodegenerative disease. We should also point out that the amyloid PET biomarker used to detect neurodegenerative disease in this study only detects signs of AD and not CTE, DLB, or other neurodegenerative disease.

Additionally, the neuropsychological and clinical characterization of veterans with a history of TBI is partly complicated by potential over-laying mood disorders either related to a relatively increased incidence of PTSD in this population (61, 62), or mood symptoms that may co-occur with neurodegenerative diseases itself including AD and CTE, both of which have been reported to have prominent mood symptoms (19, 63–66). Consistent with this literature, our head injury patients showed elevations in scales of depression and mood lability. Importantly, the presence of mood and psychiatric diagnoses did not predict ERP responses. Furthermore, the clinical and neuropsychological complexity of this population makes it possible for individuals to be diagnosed as having significant mood features potentially leading to initial under-appreciation of any potentially co-occurring neurodegenerative disease. Following the head injured group longitudinally would allow determination of whether individuals in this group eventually develop clinical neurodegenerative disease.

Neither the entire head injury group nor the subgroup with RHI exposure displayed increased rates of amyloid PET positivity or of a neurodegenerative clinical diagnosis. This is in agreement with prior literature showing studies of former professional American football players have not found increased rates of amyloid PET positivity compared to controls (67) and, as mentioned, it could also be that our sample is skewed due to being made up of individuals who presented to a memory clinic.

The presence of a cavum was not a significant predictor of head injury, neurodegenerative disease diagnosis, or amyloid PET status. While a cavum occurs in 15–50% of healthy individuals, it is thought to be present in increased frequency within the CTE population, although the exact rate is unknown (68, 69). It may be that cavums are relatively prevalent in the general population and, as such, lack specificity as a biomarker of head injury and CTE.

### Limitations

This study contained a number of limitations. While other groups examining the chronic cognitive effects of mTBI in veterans have also used self-report of TBI exposure (70), the current study does not use a validated TBI instrument and is also subject to recall bias as older participants presenting with a cognitive complaint may not have intact long-term memory. This is in part mitigated by the gathering of additional TBI/RHI history by chart review as well as by the fact that the head injuries were remote and therefore the patient's recall is more reliant on relatively intact long-term autobiographical memory as opposed to reliance on recent episodic memory which is typically more impaired in both AD or CTE (71). Furthermore, patients with unknown TBI severity were removed for sub analyses. A limitation of the current study is that there is no data on the first and most recent head injury for each participant as participants described head injuries years and decades previously and in many cases precise timing could not be determined. Finally, it is possible that the relatively normal MoCA and MMSE scores may reflect a referral bias in that patients with prior head injury history and cognitive complaints may be more likely to be referred to a memory disorders clinic at a less impaired stage than those without head injury.

Future studies could investigate *in vivo* biomarkers including tau-PET, and plasma markers of neurodegeneration in veterans with a history of TBI and/or RHI. Despite these limitations, the current study characterizes the nature of head injuries sustained in an older veteran population along with their clinical, cognitive, ERP, and structural imaging features, which may have bearing on the ultimate development of diagnostic tools for both remote head injury and subsequent neurodegenerative conditions related to head impacts.

## Conclusions

Older veterans presenting to a memory disorders clinic with cognitive complaints and history of remote head injury were less likely to have a neurodegenerative diagnosis and did not have increased rates of amyloid PET positivity or cavums by neuroimaging compared to those without head injury. Decreased amplitude of middle latency auditory evoked potentials to target and standard tones and P200 amplitude to target tones were observed in the head injury group and may reflect cognitive deficits related to remote head injury.

## Data Availability Statement

The raw data supporting the conclusions of this article will be made available by the authors, without undue reservation.

## Ethics Statement

The studies involving human participants were reviewed and approved by VA Boston Healthcare System. The patients/participants provided their written informed consent to participate in this study.

## Author Contributions

KT was responsible for design of the study, management and oversight of all aspects of the study including data entry and analysis, and interpretation as well as manuscript writing. AM and CS contributed to running the EEG/ERP paradigm, data entry, and data analysis. KS contributed efforts toward running EEG/ERP paradigm, data entry, and data analysis. PU contributed to running the EEG/ERP paradigm. AV-R and BD contributed efforts toward clinical and PET data entry and analysis as well as MRI review. RP contributed to data analysis as well as statistical support. AB contributed to study design and conceptualization, interpretation of data analysis as well as edited manuscript writing. All authors contributed to the article and approved the submitted version.

## Conflict of Interest

The authors declare that the research was conducted in the absence of any commercial or financial relationships that could be construed as a potential conflict of interest.
