# Supplementary material for: Head Injury Exposure in Veterans Presenting to Memory Disorders Clinic: An Observational Study of Clinical Characteristics and Relationship of Event-Related Potentials and Imaging Markers
Source: Front Neurol. 2021 Jun 14;12:626767. doi: 10.3389/fneur.2021.626767 (PMC8236514; doi:10.3389/fneur.2021.626767)
Supplement: Supplementary file 1 [file Data_Sheet_1.docx]

Supplementary Material

# Supplementary Figures and Tables

## Supplementary Tables

**Supplementary Table 1.** **Regression Coefficient Results for Vascular risk factors as predictors of ERP responses**

|  | **MLAEP Target AUC** | **MLAEP Standard**  **AUC** | **P50 Target Latency** | **P200 target AUC** | **P3b target AUC** |
| --- | --- | --- | --- | --- | --- |
| Constant | 92.37 (12.97) | 87.82 (5.21) | 41.09 (2.47) | 228.77(58.3) | 590.0(106.4) |
| HTN | -10.18 (16.37) | 9.14 (6.58) | -2.36 (2.09) | 32.3(73.6) | -106.4(134) |
| DM2 | -0.89 (17.38) | -5.77 (6.98) | -0.22 (3.28) | 61.89(78.15) | -113.15(142) |
| A. Fib | -40.94 (26.70) | -23.25 (10.73) | -3.88 (5.09) | 50.9(120.08) | -24.4(218.9) |
| HLD | -10.51 (16.82) | -2.53 (6.76) | 2.83 (3.16) | 55.05(75.67) | 277.1(137.9) |
| OSA | 0.15 (18.76) | -9.87 (7.54) | 2.69 (3.51) | -156.6(84.4) | -56.4(153.9) |
| Stroke | 28.18 (22.96) | -2.29 (9.23) | 0.30 (4.24) | 109.3(103.3) | 26.5(188.3) |
| CAD | 4.15 (18.43) | 7.84 (7.41) | 0.60 (3.49) | -77.6(82.9) | -53.15(151) |
| RSquared | 0.04 | 0.08 | 0.02 | 0.06 | 0.05 |
| n | 118 | 118 | 120 | 117 | 117 |
| AUC (Area Under the Curve. HTN (hypertension), DM2 (type 2 diabetes mellitus), A. fib (atrial fibrillation), HLD (hyperlipidemia), OSA (Obstructive Sleep Apnea), CAD (Coronary Artery Disease). Standard errors are reported in parentheses. | | | |  |  |

**Supplementary Table 2.** **Regression Coefficient Results for total number of vascular risk factors as predictor of ERP responses**

|  | **MLAEP Target Average AUC** | **MLAEP Standard Average AUC** | **P50 Target Latency** | **P200 target AUC** | **P3b target AUC** |
| --- | --- | --- | --- | --- | --- |
| Constant | 88.95 (11.82) | 90.03 (4.91) | 41.22 (2.25) | 261.14(54.4) | 787.5(98.3) |
| Total Vascular | -4.32 (4.51) | -1.24 (1.87) | 0.26 (0.86) | -3.13(20.7) | -22.9(37.5) |
| R-Squared | 0.01 | 0.00 | 0.00 | -0.01 | -0.005 |
| No. Observations | 121 | 121 | 123 | 120 | 120 |
| AUC (Area Under the Curve). Standard errors are reported in parentheses. | | | |  |  |
| **Supplementary Table 3.** **Regression Coefficient Results for MRI White matter lesion load as predictor of ERP responses** | | | |  |  |
|  | **MLAEP Target Average AUC** | **MLAEP Standard Average AUC** | **P50 Target Latency** | **P200 target AUC** | **P3b target AUC** |
| Constant | 83.29 (12.13) | 84.86 (4.92) | 42.92 (2.27) | 251.9(54.6) | 704.1(100.4) |
| MRI White Matter | -2.73 (8.12) | 3.14 (3.30) | -0.44 (1.53) | 2.37(36.6) | -48.7(67.2) |
| R-Squared | 0.00 | 0.01 | 0.00 | -0.01 | -.005 |
| No. Observations | 104 | 104 | 106 | 103 | 103 |
| AUC (Area Under the Curve). Standard errors are reported in parentheses. | | | |  |  |

**Supplementary Table 4.** **Regression Coefficient Results for mood measures as predictors of ERP responses**

|  | **MLAEP Target Average AUC** | **MLAEP Standard Average AUC** | **P50 Target Latency** | **P200 target AUC** | **P3b target AUC** |
| --- | --- | --- | --- | --- | --- |
| Constant | 74.99 (10.12) | 88.00 (4.19) | 40.65 (1.89) | 285.98(45.9) | 655.3(82.8) |
| PTSD | -0.41 (15.57) | -0.02 (6.45) | 0.05 (2.91) | -19.28(70.8) | 117.22(127.2) |
| Anxiety | 15.49 (21.08) | 7.96 (8.73) | 1.66 (3.99) | 128.05(95.8) | 248.74(172.5) |
| Bipolar | -14.32 (26.63) | -10.79 (11.03) | -3.89 (5.03) | -55.1(120.9) | 42.86(217.91) |
| Depression | 8.02 (15.19) | -2.15 (6.29) | 2.68 (2.85) | -80.1(69.02) | -226.5(124.4) |
| R-Squared | 0.01 | 0.01 | 0.02 | 0.03 | 0.04 |
| n | 120 | 120 | 122 | 120 | 119 |
| PTSD (post-traumatic stress disorder). Standard errors are reported in parentheses. | | | |  |  |

**Supplementary Table 5.** **Regression Coefficient Results for total number of mood disorders as predictors of ERP responses**

|  | **MLAEP Target Average AUC** | **MLAEP Standard Average AUC** | **P50 Target Latency** | **P200 target AUC** | **P3b target AUC** |
| --- | --- | --- | --- | --- | --- |
| Constant | 72.12 (9.66) | 72.12 (9.66) | 40.70 (1.82) | 264.5(44.5) | 643.3(80.6) |
| Mood Total | 7.31 (6.54) | 7.31 (6.54) | 1.06 (1.24) | -9.6(30.1) | -4.6 (54.4) |
| R-Squared | 0.01 | 0.00 | 0.00 | -0.01 | -0.01 |
| n | 121 | 121 | 123 | 120 | 120 |
| AUC (Area Under the Curve). Psych total represents summed total of presence/absence of five measures from Table 4. Standard errors are reported in parentheses. | | | |  |  |

**Supplementary Table 6.** **Regression Coefficient Results for imputed Cavum presence and MRI White Matter Lesion variables**

|  | **Head Injury** | **Neuro-degenerative Diagnosis** | **MLAEP Target Average AUC** | **MLAEP Standard Average AUC** | **P50 Target Latency** |
| --- | --- | --- | --- | --- | --- |
| Constant | 0.14 (0.22) | 0.30 (0.22) | 77.99 (11.69) | 83.36 (4.52) | 41.87 (2.10) |
| Cavum | 0.41 (0.30) | 0.12 (0.29) |  |  |  |
| MRI WML |  |  | 1.25 (8.00) | 3.16 (2.95) | -0.18 (1.40) |
| R-Squared | 0.01 | 0.01 | 0.00 | 0.01 | 0.00 |
| n | 124 | 124 | 122 | 122 | 124 |
| AUC (Area Under the Curve), MRI WML (MRI White Matter Lesions); Standard errors are reported in parentheses. | | | | | |
